# A parasite's modification of host behavior reduces predation on its host

**DOI:** 10.1002/ece3.2748

**Published:** 2017-02-05

**Authors:** John Soghigian, Linda R. Valsdottir, Todd P. Livdahl

**Affiliations:** ^1^ Department of BiologyClark UniversityWorcesterMAUSA; ^2^ Department of Environmental ScienceThe Connecticut Agricultural Experiment StationNew HavenCT06511USA

**Keywords:** *Aedes triseriatus*, *Ascogregarina*, higher‐order interaction, host–parasite interactions, mosquito, predator–prey interactions, *Toxorhynchites*

## Abstract

Parasite modification of host behavior is common, and the literature is dominated by demonstrations of enhanced predation on parasitized prey resulting in transmission of parasites to their next host. We present a case in which predation on parasitized prey is reduced. Despite theoretical modeling suggesting that this phenomenon should be common, it has been reported in only a few host–parasite–predator systems. Using a system of gregarine endosymbionts in host mosquitoes, we designed experiments to compare the vulnerability of parasitized and unparasitized mosquito larvae to predation by obligate predatory mosquito larvae and then compared behavioral features known to change in the presence of predatory cues. We exposed *Aedes triseriatus* larvae to the parasite *Ascogregarina barretti* and the predator *Toxohrynchites rutilus* and assessed larval mortality rate under each treatment condition. Further, we assessed behavioral differences in larvae due to infection and predation stimuli by recording larvae and scoring behaviors and positions within microcosms. Infection with gregarines reduced cohort mortality in the presence of the predator, but the parasite did not affect mortality alone. Further, infection by parasites altered behavior such that infected hosts thrashed less frequently than uninfected hosts and were found more frequently on or in a refuge within the microcosm. By reducing predation on their host, gregarines may be acting as mutualists in the presence of predation on their hosts. These results illustrate a higher‐order interaction, in which a relationship between a species pair (host–endosymbiont or predator–prey) is altered by the presence of a third species.

## 1 Introduction

Interactions among predators, prey, and their parasites can be complex, depending on the behavior of predator and prey organisms and on the impact of the parasite on the infected host. Impacts of parasites may be due to direct fitness consequences of parasite infection, such as the reduction in individual fitness or increase in mortality, or indirect consequences, such as reduction of competitive ability or enhanced vulnerability to predation (Aliabadi & Juliano, 2002; Hatcher, Dick, & Dunn, 2006; Hudson, Dobson, & Newborn, 1992; Murray, Cary, & Keith, 1997). Behavioral modification of hosts by parasites is one common interaction among parasites and hosts. Trophic transmission may also be increased when parasites enhance predation on prey through behavioral modification (Berdoy, Webster, & Macdonald, 2000; Lafferty & Morris, 1996; Milinski, 1985; Thomas & Poulin, 1998). Behavioral modification may be detrimental to the intermediate host, but increases the fitness of the parasite.

When a parasite is unable to complete its life cycle in a predator at the time its host is consumed, either because the predator is an ineffective host or because the parasite is not mature enough yet to infect the predator, it may be adaptive for a parasite to render its host less vulnerable to predation by that predator at that time. Despite recent theory suggesting that this predation avoidance or suppression should evolve more easily than predation enhancement (Parker, Ball, Chubb, Hammerschmidt, & Milinski, 2009), evidence for such modification is uncommon and found in very few host–parasite systems (Médoc & Beisel, 2011). One such example occurs in the early life stages of the acanthocephalan parasite *Pomphorhynchus laevis*. When infected by noninfective stages of the acanthocephalan, amphipods displayed increased antipredator behaviors associated with refuge usage; however, at a later infectious life stage of the parasite, amphipods were manipulated into behaviors that increased the risk of predation (Dianne et al., 2011). A similar effect was seen in the early life stages of two parasites that use copepods as intermediate hosts (Weinreich, Benesh, & Milinski, 2013). Examples of predation suppression come from very few host–parasite systems, all of which involve multiple hosts for the parasite (Médoc & Beisel, 2011). Adaptation in a single‐host parasite should favor predation avoidance, yet to our knowledge, this has not been demonstrated.

We used a mosquito–predator–parasite system common to the eastern United States to assess whether a single‐host parasite can reduce predation on its host. The eastern treehole mosquito, *Aedes triseriatus*, occupies treeholes and domestic water containers as larvae throughout the eastern United States (Darsie & Ward, 2005). Within these habitats, *Ae. triseriatus* cooccurs with several aquatic organisms, such as the predatory mosquito *Toxorhynchites rutilus* (Darsie & Ward, 2005)*. Toxorhynchites rutilus* feeds on aquatic insects including *Ae. triseriatus* larvae, occurring as far north as Massachusetts (Dennehy & Livdahl, 1999). *Toxorhynchites* larvae are typically ambush predators, waiting for prey to come near before attacking, and often attack when the prey use thrashing movements to move about their habitat (Russo, 1986). *Aedes triseriatus* larvae modify their behavior in the presence of *Tx. rutilus* by reducing foraging time and spending less time on the bottom of the container (Juliano & Gravel, 2002; Kesavaraju & Juliano, 2004, 2010). These responses are elicited by olfactory cues (e.g., due to chemical stimuli, Ferrari, Wisenden, & Chivers, 2010; Kesavaraju, Damal, & Juliano, 2007; or because of solid residues, Kesavaraju & Juliano, 2010).

*Ascogregarina* endosymbionts cooccur with *Ae. triseriatus and Tx. rutilus* in aquatic container habitats. This genus of largely host‐specific gregarine gut parasites primarily infects mosquitoes of the genus *Aedes* (Erthal, Soghigian, & Livdahl, 2012). *Ascogregarina* parasites infect larval mosquitoes during filter feeding and complete their life cycle within the aquatic stages of the mosquito (Chen, 1999). The host ingests the infective stage of the parasite, the oocyst, during filter feeding. Life cycle completion occurs by the pupal stage; oocysts are then released as adults eclose, and can also be released into new habitats during female oviposition (Chen, 1999). *Ascogregarina barretti* is widely distributed with its host, *Ae. triseriatus*, in natural and artificial containers where more than 70% of hosts may be naturally infected when the parasite is present (Beier & Craig, 1985). Although *A. barretti* infection has limited effects on mortality rate of its host (Copeland & Craig, 1992), the parasite causes extended female development time and smaller body size in *Ae. triseriatus*, relative to uninfected females (Walker, Poirier, & Veldman, 1987), suggesting that the parasite could regulate mosquito populations by lowering female fecundity. Based on studies showing relatively low survival cost of infection, and the ubiquitous distribution of the parasite with its host, *A. barretti* is viewed as a parasite with limited to no biocontrol possibilities in its natural host (Beier & Craig, 1985; Tseng, 2007).

To date, no behavioral effects of this parasite have been described, although alteration of larval locomotor and feeding behaviors has been reported for *Aedes aegypti* infected by mermithid nematodes. In experiments assessing whether nematodes induce behavioral shifts and predation avoidance, Wise de Valdez observed that infected larvae were less likely to browse on the bottom of a container, dived less, and were more likely to remain still underwater, but that they were just as likely as uninfected larvae to be preyed upon by *Tx. rutilus* (Wise de Valdez, 2006, 2007). Further, a related *Ascogregarina* species, *Ascogregarina taiwanensis*, has been shown to affect the competitive ability of its host mosquito *Aedes albopictus* (Aliabadi & Juliano, 2002). Together, these observations suggest that parasites of mosquito larvae can alter mosquito behavior, and that *Ascogregarina* may have the capability of modifying interactions between its host and other organisms.

No *Ascogregarina* species have been described in *Toxorhynchites* to date*. Ascogregarina* are thought to be largely host specific in nature (Chen, 1999), and although exceptions do exist (e.g., Copeland & Craig, 1992; Erthal et al., 2012), no studies to our knowledge have assessed this parasite's ability to infect the predatory *Tx. rutilus*. Assuming that *A. barretti* is host‐specific and not trophically transmitted, adaptation may have favored predation avoidance by *Ascogregarina*.

Although the effects of gregarine infection and predation on mosquitoes have been studied separately, no studies have yet explored how a combination of these factors could affect survival. Here, we test whether parasitism of *Ae. triseriatus* larvae by the gregarine parasite *A. barretti* affects vulnerability to predation by *Tx. rutilus* larvae, and attempt to determine whether these differences in vulnerability result from behavioral responses by the prey to predator presence. We present results from two experiments on *Ae. triseriatus* larvae, comparing first mortality of infected versus uninfected *Ae. triseriatus* when exposed to predation by *Tx. rutilus*, followed by a comparison of behavior and microhabitat use of infected versus uninfected *Ae. triseriatus* larvae in the presence of chemical predation cues.

## 2 Methods

### 2.1 Mosquito rearing

#### 2.1.1 Prey

We established a colony of *Ae. triseriatus* with eggs obtained from a free‐mating laboratory colony at the Connecticut Agricultural Experiment Station. We maintained the colony in an insectary at 24°C with 80% RH and a photoperiod of 16:8 (L:D) hr. To hatch larvae for experiments, we exposed egg sheets to 1 g/L of nutrient broth in distilled water for 24 hrs.

#### 2.1.2 Predator

We collected *Tx. rutilus* eggs from traps placed in North Kingston, RI, USA. The adults from these eggs were mated by anesthetizing (CO_2_) and decapitating males, gluing them to toothpicks, and connecting their abdomens to anesthetized females. The resultant eggs produced larvae that were raised individually in small plastic containers of water. Larvae of *Tx. rutilus* were fed on a diet of *Ae. triseriatus* larvae until needed in experiments by stocking *Tx. rutilus* cups with up to five larvae per day. The colony was maintained at 24°C with 80% RH and a photoperiod of 16:8 (L:D) hr.

#### 2.1.3 Parasite

We sampled field habitats of *Ae. triseriatus* in Worcester, MA, USA, known to have high infection rates of *A. barretti*. Although we have not previously detected any other *Ascogregarina* parasite within these habitats, we dissected several *Ae. triseriatus* to visually confirm parasite morphology as *A. barretti* (Beier & Craig, 1985) and we extracted DNA from oocysts shed by emerging adults and confirmed the parasite identity via PCR and subsequent sequencing of ribosomal DNA (see Erthal et al., 2012 for detailed methods). We reared field‐collected larvae to adulthood and collected oocysts shed by emerging adults. We determined the concentration of oocysts using a hemocytometer and stored the parasites at 4°C until needed for experimental use.

### 2.2 Mortality comparison

#### 2.2.1 Experimental methods

We compared the survivorship and mortality rate of prey *Ae. triseriatus* in the presence of predatory *Tx. rutilus* while infected with the parasite *A. barretti* for 10 days. To control for the effects of the parasite, we also compared mortality rate between these groups and two treatments lacking predators, either with the parasite *A. barretti* or without.

Prior to the start of the experiment, we hatched larvae and reared them for 3 days in 30‐ml petri dishes with low density (10 larvae per dish) and ample food (1 mg of brewer's yeast per day). We infected half of these petri dishes with the parasite *A. barretti* by mixing 1,000 oocysts/ml into the water of the petri dish on the first day.

Following this 3‐day period, larvae were moved into experimental microcosms. Microcosms were constructed from Reynolds Del‐Pak^®^ 16 oz. polypropylene food containers with a bottom diameter of 8.5 cm, containing 200 ml distilled water and 0.5 g of dried oak leaves cut into quarters. Additionally, each habitat was supplied with 0.5 mg of brewer's yeast every 3 days during the experiment to ensure that the prey larvae did not starve during the experiment. Our treatments were: infected prey exposed to the predator, uninfected prey exposed to the predator, infected prey alone, and uninfected prey alone. We had ten replicates for each treatment, except for the infected prey‐alone treatment, which due to a temporary oocyst shortage had only seven replicates.

At the start of the experiment, each replicate contained 12 three‐day‐old *Ae. triseriatus* mosquitoes, and where applicable, one‐second‐instar *Tx. rutilus*. The habitats were kept in an insectary at 25°C. For 10 days, the predator (where present) and leaves were removed once daily and the number of surviving prey larvae was counted. After counting, the leaves were immediately replaced and the prey larvae were allowed to acclimate to the habitat before reintroduction of the predator. After 10 days, the experiment was concluded and larvae reared for parasite colony maintenance.

During the experiment, two *Tx. rutilus* died, one from a replicate in the infected treatment and one from a replicate in the noninfected treatment. These two replicates were removed from subsequent analyses. At the end of the experiment, all of the *Tx. rutilus* larvae were dissected under a stereomicroscope and visually inspected for signs of infection by *A. barretti*. This was carried out by pulling off the head and removing the midgut to inspect for visual signs of *Ascogregarina* infection in epithelial cells (Beier & Craig, 1985).

#### 2.2.2 Analysis

All analyses were performed in R (R Core Team, 2015) using base packages, except where noted. We analyzed both cohort mortality rate and raw survivorship. We chose to assess cohort mortality as well as raw survivorship because the effect of the parasite could be subtle, and the predator is known to be efficient at capturing and consuming *Ae. triseriatus* (Bradshaw & Holzapfel, 1983; Griswold & Lounibos, 2005; Livdahl, 1979), and mortality provides a feasible measure of the effect of the parasite within an arbitrary time span in small microcosms with a limited number of larvae. For each replicate, we calculated cohort mortality rate by regressing survivorship within the replicate against time (regression details per replicate are available in the Supporting Information). The negative of the slope of this regression summarizes cohort mortality rate for a given replicate. We used a two‐way ANOVA with cohort mortality rate or raw total survivorship as a response variable and parasite treatment and predation treatment as explanatory variables, with an interaction term between the two explanatory variables. We calculated partial eta squared for each model effect (Lakens, 2013). We tested for the normality of model residuals using a Shapiro–Wilk test (Shapiro & Wilk, 1965) and for homogeneity of variances using Levene's test (Fox & Weisberg, 2010). Following detection of significant model effects, we tested for pairwise group means with a post hoc Tukey's honest significant differences test.

### 2.3 Behavioral comparison

#### 2.3.1 Experimental methods

Following our first experiment on survival differences between infected and uninfected larvae in the presence of a predator, we scored behaviors of infected and uninfected larvae in order to determine whether parasites were inducing behavioral shifts in hosts. This was carried out both with and without predator cues from *Tx. rutilus* predation on *Ae. triseriatus* larvae to see whether the presence of predator cues differentially affected parasitized and nonparasitized individuals.

We generated chemical cues by placing ten fourth instar *Ae. triseriatus* larvae in a 125‐ml flask with 60 ml of distilled water and one‐fourth instar *Tx. rutilus* larva for a period of 5 days. Each day, larvae in each flask were counted and dead or eaten larvae were replaced. At the end of the 5‐day period, larvae were removed and the water was mixed together into a single plastic container along with any solid residues left behind by the larvae. The water was frozen at −20°C for 3 months prior to the experiment to prevent degradation of chemicals released by the predator or the prey. On the day of the experiment, the frozen water was thawed at room temperature for 12 hr prior to use.

Prior to the experiment, *Ae. triseriatus* larvae were hatched and reared in low‐density petri dishes, as described previously. Half of the petri dishes were infected with the same dosage of parasites as the prior experiment, 1,000 oocysts/ml. Each petri dish was fed 1 mg of brewer's yeast every other day, and all larvae were raised for 10 days at 25°C. Larvae were reared to the fourth instar so that they would be visible in video recordings. To standardize hunger, on the last day prior to recordings being made, we withheld food from all larvae for 24 hrs.

Following the initial 10‐day period of growth, we transferred ten larvae per replicate into experimental microcosms. Microcosms were 8.5‐cm‐diameter polystyrene cups within which a 5 cm × 5 cm square of black plastic was glued, to simulate leaf litter refuge. Microcosms were filled with 150 ml of water, either full volume of distilled water, in the case of treatments without predator cues, or 120 ml of distilled water and 30 ml of water containing chemical cues and solid residues from predation. We established 10 replicates per treatment, with four treatments: infected larvae without predator cues, infected larvae with predator cues, uninfected larvae without predator cues, and uninfected larvae with predator cues.

Larvae were given five minutes to acclimate to the microcosm, after which they were recorded for 30 min. Recordings were taken from the side using a JVC GR‐D25OU while simultaneous recordings were taken from above using an Elmo P30S attached to a computer. Following recording, we scored behaviors and positions for each larva in a replicate at the beginning of each minute for thirty minutes from video assays above and to the side. We observed the first 5 s of each minute to ascertain what behavior each larvae was performing, and in which position the larvae occupied, from both videos. Behaviors were scored as follows: browsing, where the larva was being propelled through the water or along a surface due to movement of mouthparts; thrashing, where the larva was flexing laterally; and filtering/resting, where the larva's mouthparts were not in contact with the sides or the bottom of the container and the larva was not being propelled through the water by movement of mouthparts. The scores for these behaviors were based upon the descriptions in Juliano and Gravel (2002), save that we could not differentiate filter feeding from resting in our recordings due to poor resolution of larval mouthparts. We considered a score of resting to include potential filtering. Positions were scored in three areas: the top, which was within the top half of the container (about one larva's length from the surface); the bottom half of the container, which included the bottom half of the water column and the bottom of the container itself that was not covered by the refuge; and on or in the refuge, which was scored as the larva being directly on the surface of, or underneath, the black plastic refuge.

#### 2.3.2 Analysis

For each replicate, we averaged scores across the observation period such that we had the proportion of larvae performing each behavior or in each position within the replicate during the observation period. As such, we had two multivariate data sets containing observations for each replicate for each of three behaviors or positions. Two replicates (uninfected—5 and 12) were excluded from the analysis because behavioral data could not be scored due to a power failure during recording. We used separate two‐way MANOVAs to determine whether parasites and/or water‐borne chemical cues influenced behavior and position within the replicate. Following a significant MANOVA result, we examined the individual ANOVA results for each response variable independently, with a Bonferroni‐corrected α level of 0.016 for the three‐two‐way ANOVAs per MANOVA.

For each MANOVA, we tested the assumptions of multivariate normality using a multivariate Shapiro–Wilk test implemented in the package mvnormtest (Jarek, 2012) and the assumption of homogeneity of covariance matrices using Box's *M*‐test implemented in the biotools package (da Silva, 2015). When assumptions of multivariate normality were violated, we used a nonparametric randomization MANOVA (Anderson, 2001; McArdle & Anderson, 2001) from the function adonis, implemented in the package vegan (Oksanen et al., 2016). Following the randomization MANOVA, we used subsequent randomization two‐way ANOVAs due to violations of assumptions of normality (function from Mitchell & Bergmann, 2016). For each randomization test, we used 9999 permutations.

## 3 Results

### 3.1 Survival comparison

**Figure 1:**
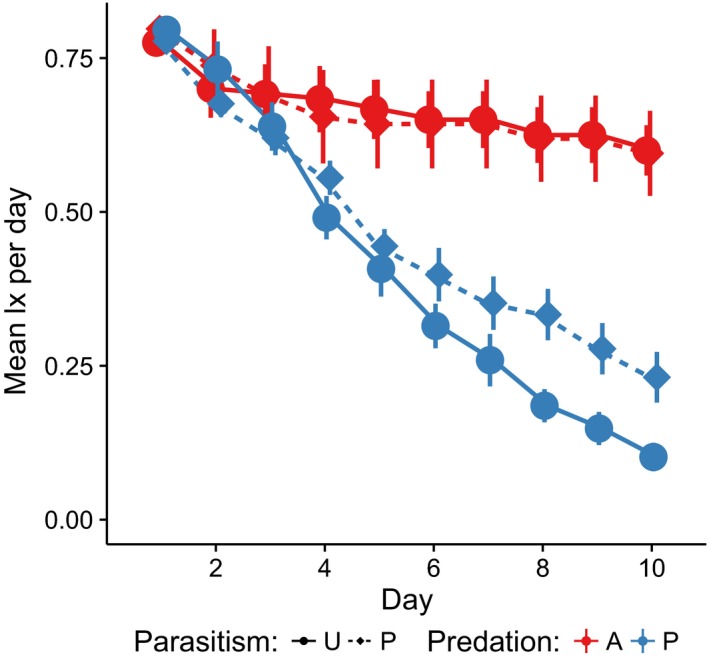
The mean l× per day over the course of the 10‐day experiment, ± one standard error. P stands for parasitized larvae (circle), while U is uninfected larvae (diamond). Predation refers to the presence (P, in blue) or absence (A, in red) of the predatory *Toxohrynchites rutilus* in the treatment

**Figure 2:**
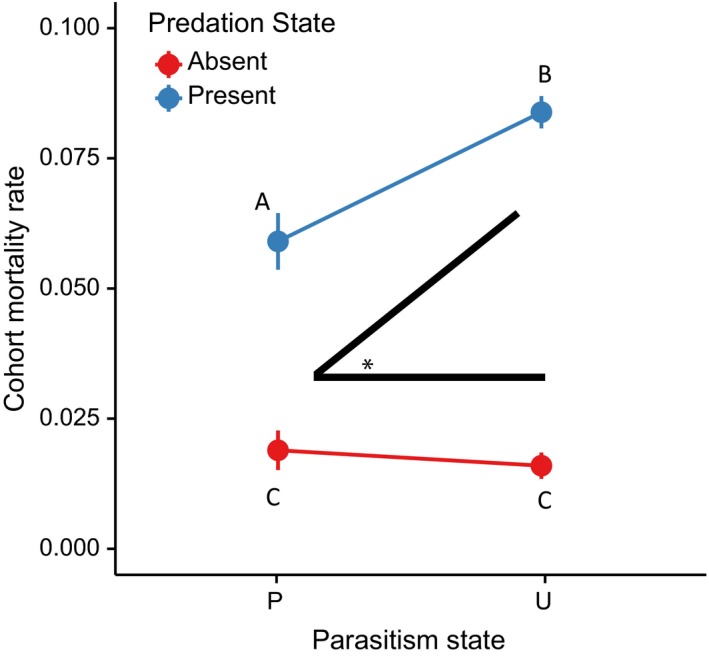
The mean cohort mortality rate of each treatment, ± one standard error. P stands for parasitized larvae, while U is uninfected larvae. Predation refers to the presence or absence of the predatory *Toxohrynchites rutilus* in the treatment. There was a significant interaction between parasitism state and predation state indicated by the black angle with asterisk (Table 1; *F*_1,31_ = 12.86, *p* = .0011). Different letters were significantly different groups based on a post hoc Tukey's honest significant difference (Table S2)

We found a significant effect of predation treatment on both survivorship and cohort mortality rate (Figures 1 and 2, Tables 1 and S1) and notably a significant interaction between parasite infection and predator presence on cohort mortality (Table 1), but not on survivorship (Table S1). Cohort mortality rate depended upon specific predation and parasitism treatment levels, while raw survivorship depended only on the presence or absence of the predator. Our post hoc Tukey's HSD following the significant interaction of parasitism and predation on cohort mortality rate indicated that while cohort mortality rate was not significantly different between parasitized and unparasitized groups when a predator was absent, cohort mortality was higher for unparasitized larvae than parasitized larvae when the predator was present (Figure 2, Table S2). Cohort mortality rate in the presence of a predator declined by approximately 30% when larvae were infected, as mean cohort mortality rate in containers exposed to predators and parasites was 0.059 (*SE* = 5.54E‐3), compared to the mean rate of 0.085 (*SE* = 3.17E‐3) for those containers exposed to predators alone (Table S2). Our two‐way models met assumptions of normality and homogeneity of variance.

**Table 1:** Two‐way analysis of variance on the effect of parasite infection and predator presence on cohort mortality rate

Effect	*df*	MS	$\eta_{p}^{2}$	*F*	*p*
Parasitism state	1	0.0004	0.085	2.996	.095
**Predation state**	**1**	**0.027**	**0.865**	**205.42**	**3.2e‐15**
**Parasitism:predation**	**1**	**0.0017**	**0.287**	**12.86**	**.0011**
Residuals	31	0.0089			

The response variable is the cohort mortality rate. Parasitism state refers to infection with *Ascogregarina barretti* or no infection, while predation state refers to presence or absence of *Toxorhynchites* in the replicate. Here we show degrees of freedom (*df*), mean squares (MS), partial eta squared ($\eta_{p}^{2}$), *F* statistics (*F*), and *p*‐values (*p*) for each effect test in the model, and we have bolded model terms that are significant at the .05 level.

### 3.2 Trophic transmission of parasite

Visual inspection of dissected *Tx. rutilus* larvae that had preyed upon infected *Ae. triseriatus* for 10 days yielded no evidence of gregarine infection in the midgut.

### 3.3 Behavior and position comparison

Due to violations of multivariate normality for both response variable sets (behavior and position), we used the function adonis in R for a nonparametric randomization MANOVA with behavioral response variables. Additionally, neither MANOVA, nor individual randomization ANOVAs that followed, showed significant interactions.

**Figure 3:**
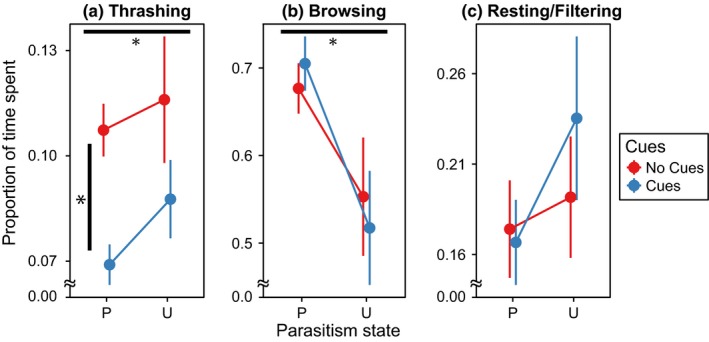
The mean proportion of larvae exhibiting specific behaviors in each treatment, ± one standard error. P stands for parasitized larvae, while U is uninfected larvae. A water state of no cues refers to the absence of chemical cues from Toxorhynchites in the replicate, while cues refer to the presence of predation cues. Horizontal black bars with asterisk indicate significant differences between parasitism states, while vertical black bars indicate significant differences between water states (Tables 2A and 3). See methods for details on scoring of behaviors

We found a significant effect of parasitism on the behavior of larvae, but in the overall test of all response variables, we found no effect of predation cues (Figure 3, Table 2A). Following this result, we used individual randomization ANOVAs to determine whether all behaviors, or only certain behaviors, were being affected by our treatments. Using an alpha level of 0.016, we found significant effects of parasitism and predator cues on thrashing behavior (Table 3). Larvae thrashed less while infected; larvae also thrashed less in the presence of *Tx. rutilus* chemical cues (Figure 3). Additionally, we found that a larger proportion of infected larvae were scored as browsing than uninfected larvae (Table 3). Because of our use of a corrected alpha level, we found no significant difference between resting/browsing between parasitism states despite a *p* value below 0.5.

**Table 2:** Two multivariate analysis of variances on the effect of chemical cues and parasitism on larval behavior and position

Effect	*df*	MS	*F*	*p*
A. Response variables: locomotion and feeding behaviors				
Chemical cues	1	0.019	1.68	.19
**Parasitism status**	**1**	**0.081**	**7.16**	**.0039**
Cues:parasitism	1	0.012	1.09	.31
Residuals	34	0.011		
B. Response variables: position in microcosm				
Chemical cues	1	0.001	0.068	.87
**Parasitism status**	**1**	**0.32**	**35.18**	**.0001**
Cues:parasitism	1	0.009	0.39	.56
Residuals	35	0.0089		

Chemical cues indicate whether predatory cues were present or absent, while parasitism status refers to larvae infected with *Ascogregarina barretti* or uninfected. Here we show degrees of freedom (*df*), mean squares (MS), *F* statistics (*F*), and *p*‐values (*p*) for each effect test in the model, and we have bolded model terms that are significant at the .05 level.

**Table 3:** Randomization analysis of variances showing each effect and each response variable based on behavior

Effect	Behavior	*df*	MS	*F*	*p*
Chemical cues	Resting	1	0.003	0.40	.55
	Browsing	1	0.000	0.015	.90
	**Thrashing**	**1**	**0.012**	**15.88**	**.0007**
Infection status	Resting	1	0.042	4.82	.032
	**Browsing**	**1**	**0.088**	**9.44**	**.0037**
	**Thrashing**	**1**	**0.006**	**8.03**	**.0087**
Cues:parasitism	Resting	1	0.007	0.84	.383
	Browsing	1	0.011	1.18	.295
	Thrashing	1	0.000	0.15	.706
Residuals	Resting	34	0.009		
	Browsing	34	0.009		
	Thrashing	34	0.001		

We have grouped results from each behavior according to the different effects for easier comparison. The response variable for each ANOVA is the proportion of larvae exhibiting one of the three behaviors. Chemical cues indicate whether predatory cues were present or absent, while parasitism status refers to larvae infected with *Ascogregarina barretti* or uninfected. Behavior indicates which response value was used for the particular randomization ANOVA. We show degrees of freedom (*df*), mean squares (MS), *F* statistics (*F*), and *p*‐values (*p*) for each effect test in the model, and we have bolded model terms that are significant at the .016 level.

**Figure 4:**
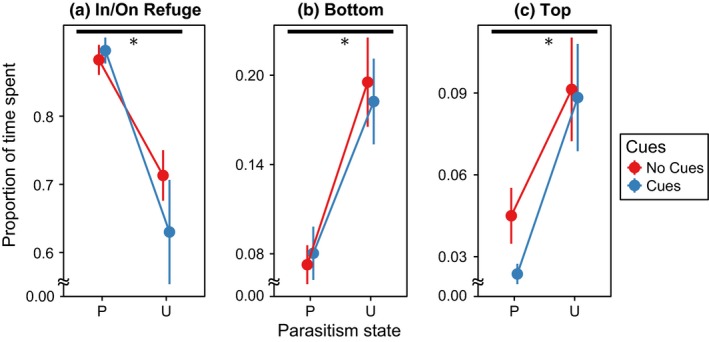
The mean proportion of larvae in specific positions in each treatment, ± one standard error. P stands for parasitized larvae, while U is uninfected larvae. A water state of no cues refers to the absence of chemical cues from Toxorhynchites in the replicate, while cues refer to the presence of predation cues. Horizontal black bars with asterisk indicate significant differences between parasitism states, while vertical black bars indicate significant differences between water states (Tables 2B and 4). See methods for details on scoring of positions

We also found a significant effect of parasitism on larval position within the container, but no effect of chemical cues on larval position (Figure 4, Table 2B). Subsequent randomization ANOVAs showed that parasitized larvae were found more often in or near the refuge than in other positions, relative to uninfected larvae (Figure 4, Table 4).

**Table 4:** Randomization analysis of variances showing each effect and each response variable based on position

Effect	Position	*df*	MS	*F*	*p*
Chemical cues	Top	1	0.001	0.29	.60
	Bottom	1	0.000	0.033	.85
	In/on refuge	1	0.0002	0.023	.88
Infection status	**Top**	**1**	**0.0344**	**16.91**	**.0001**
	**Bottom**	**1**	**0.1506**	**33.31**	**<.0001**
	**In/on refuge**	**1**	**0.3296**	**40.81**	**<.0001**
Cues:parasitism	Top	1	0.007	0.84	.3
	Bottom	1	0.011	1.18	.30
	In/on refuge	1	0.000	0.15	.71
Residuals	Top	34	0.0020		
	Bottom	35	0.0045		
	In/on refuge	35	0.0080		

We have grouped results from each position according to the different effects for easier comparison. The response variable for each ANOVA is the proportion of larvae in one of the three positions. Chemical cues indicate whether predatory cues were present or absent, while parasitism status refers to larvae infected with *Ascogregarina barretti* or uninfected. Behavior indicates which response value was used for the particular randomization ANOVA. We show degrees of freedom (*df*), mean squares (MS), *F* statistics (*F*), and *p*‐values (*p*) for each effect test in the model, and we have bolded model terms that are significant at the .016 level.

## 4 Discussion

Consistent with past literature (Copeland & Craig, 1992; Walker et al., 1987), we found no significant effect of this parasite alone on cohort mortality rate, but *Ae. triseriatus* parasitized by *A. barretti* had lower cohort mortality when exposed to *Tx. rutilus* predators than did uninfected *Ae. triseriatus* (Table 1; Figure 2). It is not altogether surprising that we found no significant difference in survivorship at the end of 10 days between parasitism treatments exposed to predators; 10 days was an arbitrary time interval, our microcosms were small and contained a limited number of prey, and *Tx. rutilus* is an extremely efficient predator that is known to prey heavily on *Ae. triseriatus* throughout their overlapping ranges (Bradshaw & Holzapfel, 1983; Griswold & Lounibos, 2005; Lounibos, Escher, Nishimura, & Juliano, 1997). However, that we detected a difference in mortality rate suggests that infected *Ae. triseriatus* were consumed at a lower rate and thus the parasite reduces predation on its host.

Our results from the visual inspection of dissected *Tx. rutilus* larvae suggest that *A. barretti* is unable to infect the predator through trophic transmission. If this is indeed the case, then there is no selective pressure for the parasite to reach the predator, and in fact, there would be selective pressure on the parasite to avoid host predation, as *Tx. rutilus* represents a dead end for the parasite.

Our behavioral assays provide some insight into how infected larvae are consumed at a rate different from uninfected larvae. Infected larvae spent significantly more time near the refuge than uninfected larvae, both in clean water and in the presence of chemical cues from predation. In natural environments, spending more time near refuges could make it harder for a predator to detect and capture infected individuals due to visual and physical obstruction by leaf litter and other debris in the water. These results are consistent with previous findings on enhanced predation avoidance, in which amphipods infected by *P. laevis* increased refuge use in the presence of a predator (Dianne, Perrot‐Minnot, Bauer, Guvenatam, & Rigaud, 2014). Although refuges are thought to reduce the overall number of encounters between predators and prey, this phenomenon may be system‐specific. For instance, habitat complexity did not deter *Tx. rutilus* predation on *A. albopictus*, nor did it deter predation on *Ae. triseriatus* by *Corethrella appendiculata* (Alto, Griswold, & Lounibos, 2005). However, because *A. albopictus* is more susceptible to predation by *Tx. rutilus* than *Ae. triseriatus* (Griswold & Lounibos, 2005), it is possible that habitat complexity could favor *Ae. triseriatus* despite not favoring *A. albopictus*. Furthermore, Edgerly, Willey, and Livdahl (1999) found that habitat complexity reduced intraguild predation in *Ae. triseriatus*, suggesting that in certain predatory interactions between mosquitoes, habitat complexity plays an important role in larval survival. Refuge use thus could be a factor explaining the discrepancy in cohort mortality rate, but further studies would be needed to assess the degree to which refuges influence *Tx. rutilus* predation on *Ae. triseriatus*.

Infected larvae browsed more, and qualitatively filtered/rested less than uninfected larvae, suggesting a change in the type of foraging behavior used by infected mosquitoes. Browsing and filtering are considered medium‐risk activities, while resting is a low‐risk behavior (Juliano & Reminger, 1992). Thus, our need to combine resting and filtering makes it difficult to determine precisely how parasitism modified risk in terms of foraging behaviors alone, because while parasitism led to an increase in proportion of browsing larvae, uninfected larvae could have been filtering more than infected larvae and thus have an equal relative risk of predation from these behaviors.

We found no significant effect of chemical cues on either browsing or resting/filtering, contrary to what has been reported in the literature (Juliano & Gravel, 2002; Kesavaraju & Juliano, 2004, 2010). However, at least for resting/filtering, this may be due to our lack of resolution between filtering and resting; if, for instance, larvae ceased filtering at the surface in chemical cues and instead were resting, they would be scored as resting/filtering under both treatments. Thus, although we found no effect of chemical cues on foraging behaviors, this may be a consequence of our methods. Alternatively, it is possible that because the mosquitoes we used were from Connecticut, near the range limits of *Tx. rutilus* (Darsie & Ward, 2005), adaptation by *Ae. triseriatus* to *Tx. rutilus* predation cues may be incomplete, as *Ae. triseriatus* shows geographic variation in response to *Tx. rutilus* predation (Juliano & Reminger, 1992).

Parasitized larvae exposed to *Tx. rutilus* chemical cues thrashed the least of all experimental treatments. Thrashing behaviors are considered the highest risk behaviors (Juliano & Reminger, 1992), and *Ae. triseriatus* has adapted to thrash less in the presence of predation cues (Juliano & Gravel, 2002; Kesavaraju & Juliano, 2010). Our results of an effect of predation cues on thrashing behavior are consistent with results of other studies (Juliano & Gravel, 2002; Kesavaraju & Juliano, 2004, 2010; Kesavaraju et al., 2007).

Our findings conform with the hypothesis that an endosymbiont of a prey host that cannot continue its life cycle within a predator might minimize its host's risk of predation. However, it is unclear from these experiments alone whether the alterations in prey behavior resulted from direct parasite manipulation or indirectly through other processes mediated by the parasite, the end result of which was a host better able to avoid predation. The observed decrease in thrashing, increase in time spent near the refuge, and increase in browsing behavior may be due to the parasitized larva's increased need to forage; any amount of time spent foraging is time not spent thrashing, and as the refuge provides increased surface area in the habitat, the larva may spend more time foraging on that surface. Were this not the case, and were the parasite responding specifically the presence of a predator, we might expect an interaction between predation cues and the influence of the parasite on position or predation cues. In the absence of such an interaction, it seems plausible that the predation suppression observed here is an indirect effect of the parasite. Despite this, selection could act on indirect effects of the parasite and from an ecological perspective the exact mechanism for the behavioral response is less important than the reduced cohort mortality of parasitized larvae.

To our knowledge, this study is the first to demonstrate a single‐host parasite reducing predation rates on its host. Furthermore, our findings suggest the potential for a context‐specific mutualistic relationship in which *A. barretti* and *Ae. triseriatus* benefit one another in the presence of *Tx. rutilus*. This higher‐order interaction highlights the importance of considering community‐level interactions among species when assessing relationships. *Ascogregarina* are considered parasites, but in this case produce context‐specific survival benefits. Although further studies that more precisely assess fitness consequences of infection in the presence of the predator must be undertaken (e.g., with fecundity measurements), our results suggest that the identification of ecological interactions based on isolated pairwise species effects on one another may be insufficient for the mosquito–gregarine system presented here.

## Conflict of Interest

None declared.

## Data Accessibility

Linear models used in cohort mortality analyses and behavioral data accompany this article as an electronic supplement.

## References

[ece32748-bib-0001] Aliabadi, B. W. , & Juliano, S. A. (2002). Escape from gregarine parasites affects the competitive interactions of an invasive mosquito. Biological Invasions, 4, 283–297. 19777120 10.1023/A:1020933705556 PMC2748405

[ece32748-bib-0002] Alto, B. W. , Griswold, M. W. , & Lounibos, L. P. (2005). Habitat complexity and sex‐dependent predation of mosquito larvae in containers. Oecologia, 146, 300–310. doi: 10.1007/s00442‐005‐0198‐x 16041612 10.1007/s00442-005-0198-x PMC3334868

[ece32748-bib-0003] Anderson, M. J. (2001). A new method for non‐parametric multivariate analysis of variance. Austral Ecology, 26, 32–46. doi: 10.1111/j.1442‐9993.2001.01070.pp.x

[ece32748-bib-0004] Beier, J. C. , & Craig, G. B. (1985). Gregarine parasites of mosquitoes ln Laird M., Miles J. W. (Eds.), Integrated mosquito control methodologies (pp. 167–184). London, UK: Academic Press.

[ece32748-bib-0005] Berdoy, M. , Webster, J. P. , & Macdonald, D. W. (2000). Fatal attraction in rats infected with *Toxoplasma gondii* . Proceedings of the Royal Society of London. Series B: Biological Sciences, 267, 1591–1594. doi: 10.1098/rspb.2000.1182 11007336 10.1098/rspb.2000.1182 PMC1690701

[ece32748-bib-0006] Bradshaw, W. E. , & Holzapfel, C. M. (1983). Predator‐mediated, non‐equilibrium coexistence of tree‐hole mosquitoes in southeastern North America. Oecologia, 57, 239–256. doi: 10.1007/BF00379586 10.1007/BF00379586 28310181

[ece32748-bib-0007] Chen, W. J. (1999). The life cycle of *Ascogregarina taiwanensis* (Apicomplexa:Lecudinidae). Parasitology Today, 15, 153–156. 10322337 10.1016/s0169-4758(99)01418-0

[ece32748-bib-0008] Copeland, R. S. , & Craig, G. B. (1992). Interspecific competition, parasitism, and predation affect development of *Aedes hendersoni* and *A. triseriatus* (Diptera: Culicidae) in artificial treeholes. Annals of the Entomological Society of America, 85, 154–163.

[ece32748-bib-0009] Darsie, R. F. J. , & Ward, R. A. (2005). Distribution of the mosquitoes of North America, north of Mexico. Mosquito Systematics Supplement, 1, 1–313.

[ece32748-bib-0010] Dennehy, J. , & Livdahl, T. P. (1999). First record of *Toxorhynchites rutilus* (Diptera: Culicidae) in Massachusetts. Journal of the American Mosquito Control Association, 15, 423–424. 10480135

[ece32748-bib-0011] Dianne, L. , Perrot‐Minnot, M.‐J. , Bauer, A. , Gaillard, M. , Léger, E. , & Rigaud, T. (2011). Protection first then facilitation: A manipulative parasite modulates the vulnerability to predation of its intermediate host according to its own developmental stage. Evolution, 65, 2692–2698. doi: 10.1111/j.1558‐5646.2011.01330.x 21884065 10.1111/j.1558-5646.2011.01330.x

[ece32748-bib-0012] Dianne, L. , Perrot‐Minnot, M.‐J. , Bauer, A. , Guvenatam, A. , & Rigaud, T. (2014). Parasite‐induced alteration of plastic response to predation threat: Increased refuge use but lower food intake in *Gammarus pulex* infected with the acanothocephalan *Pomphorhynchus laevis* . International Journal for Parasitology, 44, 211–216. doi: 10.1016/j.ijpara.2013.11.001 24291320 10.1016/j.ijpara.2013.11.001

[ece32748-bib-0013] Edgerly, J. S. , Willey, M. S. , & Livdahl, T. (1999). Intraguild predation among larval treehole mosquitoes, *Aedes albopictus*,* Ae. aegypti*, and *Ae. triseriatus* (Diptera: Culicidae), in laboratory microcosms. Journal of Medical Entomology, 36, 394–399. 10337114 10.1093/jmedent/36.3.394

[ece32748-bib-0014] Erthal, J. A. , Soghigian, J. S. , & Livdahl, T. (2012). Life cycle completion of parasite *Ascogregarina taiwanensis* (Apicomplexa: Lecudinidae) in non‐native host *Ochlerotatus japonicus* (Diptera: Culicidae). Journal of Medical Entomology, 49, 1109–1117. doi: 10.1603/ME12018 23025193 10.1603/me12018

[ece32748-bib-0015] Ferrari, M. C. O. , Wisenden, B. D. , & Chivers, D. P. (2010). Chemical ecology of predator–prey interactions in aquatic ecosystems: A review and prospectus. Canadian Journal of Zoology, 88, 698–724. doi: 10.1139/Z10‐029

[ece32748-bib-0016] Fox, J. , & Weisberg, H. (2010). An R companion to applied regression (2nd ed.). Thousand Oaks, CA: SAGE Publications Inc.

[ece32748-bib-0017] Griswold, M. W. , & Lounibos, L. P. (2005). Does differential predation permit invasive and native mosquito larvae to coexist in Florida? Ecological Entomology, 30, 122–127. doi: 10.1111/j.0307‐6946.2005.00671.x 22532705 10.1111/j.0307-6946.2005.00671.x PMC3332128

[ece32748-bib-0018] Hatcher, M. J. , Dick, J. T. A. , & Dunn, A. M. (2006). How parasites affect interactions between competitors and predators. Ecology Letters, 9, 1253–1271. doi: 10.1111/j.1461‐0248.2006.00964.x 17040328 10.1111/j.1461-0248.2006.00964.x

[ece32748-bib-0019] Hudson, P. J. , Dobson, A. P. , & Newborn, D. (1992). Do parasites make prey vulnerable to predation? Red grouse and parasites. Journal of Animal Ecology, 61, 681–692. doi: 10.2307/5623

[ece32748-bib-0020] Jarek, S. (2012). mvnormtest: Normality test for multivariate variables [WWW Document]. Retrieved from http://CRAN.R-project.org/package=mvnormtest

[ece32748-bib-0021] Juliano, S. A. , & Gravel, M. E. (2002). Predation and the evolution of prey behavior: An experiment with tree hole mosquitoes. Behavioral Ecology, 13, 301–311. doi: 10.1093/beheco/13.3.301

[ece32748-bib-0022] Juliano, S. A. , & Reminger, L. (1992). The relationship between vulnerability to predation and behavior of larval treehole mosquitoes: Geographic and ontogenetic differences. Oikos, 63, 465. doi: 10.2307/3544974

[ece32748-bib-0023] Kesavaraju, B. , Damal, K. , & Juliano, S. A. (2007). Threat‐sensitive behavioral responses to concentrations of water‐borne cues from predation. Ethology, 113, 199–206. doi: 10.1111/j.1439‐0310.2006.01317.x 17440601 10.1111/j.1439-0310.2006.01317.x PMC1852435

[ece32748-bib-0024] Kesavaraju, B. , & Juliano, S. A. (2004). Differential behavioral responses to water‐borne cues to predation in two container‐dwelling mosquitoes. Annals of the Entomological Society of America, 97, 194–201. 17710216 10.1603/0013-8746(2004)097[0194:dbrtwc]2.0.co;2 PMC1950130

[ece32748-bib-0025] Kesavaraju, B. , & Juliano, S. A. (2010). Nature of predation risk cues in container systems: Mosquito responses to solid residues from predation. Annals of the Entomological Society of America, 103, 1038–1045. doi: 10.1603/AN10007 22740721 10.1603/AN10007 PMC3381358

[ece32748-bib-0026] Lafferty, K. D. , & Morris, A. K. (1996). Altered behavior of parasitized killifish increases susceptibility to predation by bird final hosts. Ecology, 77, 1390–1397. doi: 10.2307/2265536

[ece32748-bib-0027] Lakens, D. (2013). Calculating and reporting effect sizes to facilitate cumulative science: A practical primer for t‐tests and ANOVAs. Frontiers in Psychology, 4, 1–12. doi: 10.3389/fpsyg.2013.00863 24324449 10.3389/fpsyg.2013.00863 PMC3840331

[ece32748-bib-0028] Livdahl, T. P. (1979). Evolution of handling time: The functional response of a predator to the density of sympatric and allopatric strains of prey. Evolution, 33, 765–768. doi: 10.2307/2407798 10.1111/j.1558-5646.1979.tb04728.x 28563944

[ece32748-bib-0029] Lounibos, L. P. , Escher, R. L. , Nishimura, N. , & Juliano, S. A. (1997). Long‐term dynamics of a predator used for biological control and decoupling from mosquito prey in a subtropical treehole ecosystem. Oecologia, 111, 189–200. doi: 10.1007/s004420050225 10.1007/s004420050225 28307994

[ece32748-bib-0030] McArdle, B. H. , & Anderson, M. J. (2001). Fitting multivariate models to community data: A comment on distance‐based redundancy analysis. Ecology, 82, 290–297. doi: 10.2307/2680104

[ece32748-bib-0031] Médoc, V. , & Beisel, J.‐N. (2011). When trophically‐transmitted parasites combine predation enhancement with predation suppression to optimize their transmission. Oikos, 120, 1452–1458. doi: 10.1111/j.1600‐0706.2011.19585.x

[ece32748-bib-0032] Milinski, M. (1985). Risk of predation of parasitized sticklebacks (*Gasterosteus aculeatus* L.) under competition for food. Behaviour, 93, 203–216. doi: 10.1163/156853986X00883

[ece32748-bib-0033] Mitchell, A. , & Bergmann, P. J. (2016). Thermal and moisture habitat preferences do not maximize jumping performance in frogs. Functional Ecology, 30, 733–742. doi: 10.1111/1365‐2435.12535

[ece32748-bib-0034] Murray, D. L. , Cary, J. R. , & Keith, L. B. (1997). Interactive effects of sublethal nematodes and nutritional status on snowshoe hare vulnerability to predation. Journal of Animal Ecology, 66, 250–264. doi: 10.2307/6026

[ece32748-bib-0035] Oksanen, J. , Blanchet, F. G. , Kindt, R. , Legendre, P. , Minchin, P. R. , O'Hara, R. B. , … Szoecs, E. (2016). vegan: Community ecology package.

[ece32748-bib-0036] Parker, G. A. , Ball, M. A. , Chubb, J. C. , Hammerschmidt, K. , & Milinski, M. (2009). When should a trophically transmitted parasite manipulate its host? Evolution, 63, 448–458. doi: 10.1111/j.1558‐5646.2008.00565.x 19154358 10.1111/j.1558-5646.2008.00565.x

[ece32748-bib-0037] R Core Team (2015). R: A language and environment for statistical computing. Vienna, Austria: R Foundation for Statistical Computing.

[ece32748-bib-0410] Russo, R. (1986). Comparison of Predatory Behavior in Five Species of Toxorhynchites (Diptera: Culicidae). Annals of the Entomological Society of America, 79, 715–722.

[ece32748-bib-0038] Shapiro, S. S. , & Wilk, M. B. (1965). An analysis of variance test for normality (complete samples). Biometrika, 52, 591–611. doi: 10.2307/2333709

[ece32748-bib-0039] da Silva, A. R. (2015). biotools: Tools for biometry and applied statistics in agricultural science.

[ece32748-bib-0040] Thomas, F. , & Poulin, R. (1998). Manipulation of a mollusc by a trophically transmitted parasite: Convergent evolution or phylogenetic inheritance? Parasitology, 116(Pt 5), 431–436. 9614326 10.1017/s003118209800239x

[ece32748-bib-0041] Tseng, M. (2007). Ascogregarine parasites as possible biocontrol agents of mosquitoes. Journal of the American Mosquito Control Association, 23, 30–34. 17853595 10.2987/8756-971X(2007)23[30:APAPBA]2.0.CO;2

[ece32748-bib-0042] Walker, E. D. , Poirier, S. J. , & Veldman, W. T. (1987). Effects of *Ascogregarina baretti* (Eugrgarinida: Lecudinidae) infection on emergence success, development time, and size of *Aedes triseriatus* (Diptera: Culicidae) in microcosms and tires. Journal of Medical Entomology, 24, 303–309. 3108509 10.1093/jmedent/24.3.303

[ece32748-bib-0043] Weinreich, F. , Benesh, D. P. , & Milinski, M. (2013). Suppression of predation on the intermediate host by two trophically‐transmitted parasites when uninfective. Parasitology, 140, 129–135. doi: 10.1017/S0031182012001266 22906915 10.1017/S0031182012001266

[ece32748-bib-0044] Wise de Valdez, M. R. (2006). Parasitoid‐induced behavioral alterations of *Aedes aegypti* mosquito larvae infected with mermithid nematodes (Nematoda: Mermithidae). Journal of Vector Ecology, 31, 344–354. doi: 10.3376/1081‐1710(2006) 31[344:PBAOAA]2.0.CO;2 17249352 10.3376/1081-1710(2006)31[344:pbaoaa]2.0.co;2

[ece32748-bib-0045] Wise de Valdez, M. R. (2007). Predator avoidance behavior of *Aedes aegypti* mosquito larvae infected with mermithid nematodes (Nematoda: Mermithidae). Journal of Vector Ecology, 32, 150–153. doi: 10.3376/1081‐1710(2007) 32[150:PABOAA]2.0.CO;2 17633436 10.3376/1081-1710(2007)32[150:paboaa]2.0.co;2

