# Supplementary material for: A parasite's modification of host behavior reduces predation on its host
Source: Ecol Evol. 2017 Feb 5;7(5):1453–61. doi: 10.1002/ece3.2748 (PMC5330890; doi:10.1002/ece3.2748)
Supplement: Supplementary file 1 [file ECE3-7-1453-s001.docx]

SUPPLEMENTAL MATERIALS

soghigian_et_al_parapred.xlsx contains cohort mortality information and behavioral proportions analyzed in Soghigian et al.

| Supplemental Table 2: The posthoc Tukey’s Honest Significant Difference results following a two-way ANOVA on the effect of parasite infection and predator presence on cohort mortality rate (see Table 1, Figure 1). | | | | |
| --- | --- | --- | --- | --- |
| Treatment Combinations | Difference | Lower Bound | Upper Bound | p-adj |
| Parasite Absent, Predator Present with  Parasite Present, Predator Present | 0.03 | 0.01 | 0.04 | 3.34E-04 |
| Parasite Present, Predator Absent with  Parasite Present, Predator Present | -0.04 | -0.06 | -0.03 | 4.00E-07 |
| Parasite Absent, Predator Absent with  Parasite Present, Predator Present | -0.04 | -0.06 | -0.03 | 0.00E+00 |
| Parasite Present, Predator Absent with  Parasite Absent, Predator Present | -0.07 | -0.08 | -0.05 | 0.00E+00 |
| Parasite Absent, Predator Absent with  Parasite Absent, Predator Present | -0.07 | -0.08 | -0.05 | 0.00E+00 |
| Parasite Absent, Predator Absent with  Parasite Present, Predator Absent | 0.00 | -0.02 | 0.01 | 0.95 |

| Supplemental Table 1: Two-way Analysis of Variance on the effect of parasite infection and predator presence on larval survivorship. | | | | | |
| --- | --- | --- | --- | --- | --- |
| Effect | Df | MS | η^2^_p_ | F | P |
| Parasitism state | 1 | 0.88 | 0.011 | 0.373 | 0.546 |
| **Predation state** | **1** | **238.70** | **0.764** | **100.582** | **2.99e-11** |
| Parasitism:Predation | 1 | 5.59 | 0.071 | 2.356 | 0.135 |
| Residuals | 31 | 0.0089 |  |  |  |
| The response variable is the raw larval survivorship after 10 days. Parasitism state refers to infection with *A. barretti* or no infection, while predation state refers to presence or absence of *Toxorhynchites* in the replicate. Here we show degrees of freedom (Df), mean squares (MS), partial eta squared (η^2^_p_), F statistics (F) and P-values (P) for each effect test in the model, and we have bolded model terms that are significant at the 0.05 level. | | | | | |
